# Association Between the Preoperative Triglyceride–Glucose Index and Acute Kidney Injury in Patients With Chronic Kidney Disease Undergoing Cardiac Surgery

**DOI:** 10.31083/RCM28110

**Published:** 2025-06-17

**Authors:** Zhen Zhang, Yanwen Jiang, Zhe Luo, Yi Fang, Xiaoqiang Ding, Wuhua Jiang

**Affiliations:** ^1^Department of Nephrology, Zhongshan Hospital, Fudan University, 200032 Shanghai, China; ^2^Shanghai Institute of Kidney and Dialysis, 200032 Shanghai, China; ^3^Department of Nursing, Zhongshan Hospital, Fudan University, 200032 Shanghai, China; ^4^Cardiac Intensive Care Center, Zhongshan Hospital, Fudan University, 200032 Shanghai, China; ^5^Shanghai Key Laboratory of Kidney and Blood Purification, 200032 Shanghai, China; ^6^Shanghai Clinical Medical Center for Kidney Disease, 200032 Shanghai, China

**Keywords:** acute kidney injury, cardiac surgery, kidney dysfunction, TyG, insulin resistance

## Abstract

**Background:**

Acute kidney injury (AKI) is a major complication of cardiac surgery, particularly in patients with pre-existing chronic kidney disease (CKD), who are at higher risk due to their compromised renal function. This study investigated the association between the triglyceride–glucose (TyG) index, a marker of insulin resistance, and postoperative AKI in CKD patients undergoing cardiac surgery to enhance risk stratification and perioperative management.

**Methods:**

This retrospective study included 542 patients with impaired renal function (estimated glomerular filtration rate (eGFR) 15–60 mL/min/1.73 m^2^) undergoing cardiac surgery from January 2018 to December 2019. The TyG index was calculated as Ln(fasting triglycerides [mg/dL] × fasting blood glucose [mg/dL]/2), and outcomes were defined as postoperative AKI (per Kidney Disease: Improving Global Outcomes (KDIGO) criteria), in-hospital mortality, and length of hospital stay. Multivariate logistic regression and subgroup analyses assessed the association between TyG and these endpoints.

**Results:**

Among the 542 patients, 55.7% developed AKI, and the in-hospital mortality rate was 7.6%. In the multivariate regression analysis, the odds ratio for AKI with each unit increase in LnTyG was 0.43 (95% CI 0.02–8.70, *p* = 0.579), while in the standardized TyG, it was 0.96 (95% CI 0.77–1.21, *p* = 0.754). Subgroup analyses, stratified by age, sex, CKD stage, and diabetes status, revealed no significant associations across all strata (all *p* for interaction > 0.05).

**Conclusion:**

The TyG index is not significantly associated with AKI or prognosis after cardiac surgery in patients with kidney dysfunction. Further studies are needed to elucidate the role of insulin resistance in the pathogenesis of AKI.

## 1. Introduction

Acute kidney injury (AKI) is a severe complication with a high incidence among patients undergoing cardiac surgery, particularly those with pre-existing chronic kidney disease (CKD), who are at greater risk due to their reduced renal reserve and heightened susceptibility to perioperative stressors. AKI significantly elevates postoperative mortality, prolongs hospital stay, and accelerates progression to end-stage kidney disease [1, 2, 3]. Based on the limited studies available, the incidence of AKI in CKD patients undergoing cardiac surgery is reported to be approximately 50% [4], highlighting the urgent need for effective risk stratification and management strategies to mitigate its impact.

Insulin resistance (IR) is an early metabolic alteration in CKD patients [5]. It becomes apparent when the glomerular filtration rate is still within the normal range and is almost universal in those who reach the end stage of kidney failure [6]. IR plays a crucial role in the pathophysiology of various metabolic and cardiovascular diseases [7]. The mechanisms underlying IR involve complex interactions between metabolic and inflammatory pathways, which can exacerbate renal injury, particularly in patients undergoing cardiac surgery [8]. During cardiac surgery, patients often experience hemodynamic instability, oxidative stress, and inflammation, all of which can contribute to the development of AKI [9]. IR can further impair renal perfusion and promote renal tubular damage through various mechanisms, including endothelial dysfunction [10], increased sympathetic activity [11], and activation of the renin-angiotensin-aldosterone system [12] (RAAS)​.

The triglyceride-glucose (TyG) index is a surrogate marker for insulin resistance [13], calculated as Ln(fasting triglycerides [mg/dL] × fasting blood glucose [mg/dL]/2). Compared to traditional methods such as homeostatic model assessment of IR, the TyG index offers several advantages: it is simpler to calculate, requires only routine biochemical measurements, and has demonstrated reliability in diverse clinical settings. These attributes make it particularly suitable for large-scale clinical studies in patient populations where advanced testing methods are impractical. Previous studies have demonstrated that a higher TyG index is associated with an increased risk of various renal outcomes, including AKI, in different patient populations. Studies have shown that elevated TyG index levels are linked to a higher incidence of AKI in critically ill patients [14, 15], those undergoing coronary revascularization [16], and patients with acute myocardial infarction​ [17]​.

Despite growing evidence linking the TyG index to adverse outcomes in the general population and patients with cardiovascular diseases, its predictive value in CKD patients undergoing cardiac surgery remains unexplored. Previous studies have largely focused on the general population or patients with acute myocardial infarction and coronary artery diseases, leaving a significant knowledge gap in this high-risk surgical cohort. This study aims to investigate whether the TyG index can independently predict AKI in CKD patients undergoing cardiac surgery. We hypothesize that the TyG index is associated with an increased risk of AKI and could serve as a valuable tool for perioperative risk stratification in this vulnerable population.

## 2. Materials and Methods

### 2.1 Patients and Inclusion/Exclusion Criteria

**Fig. 1.**
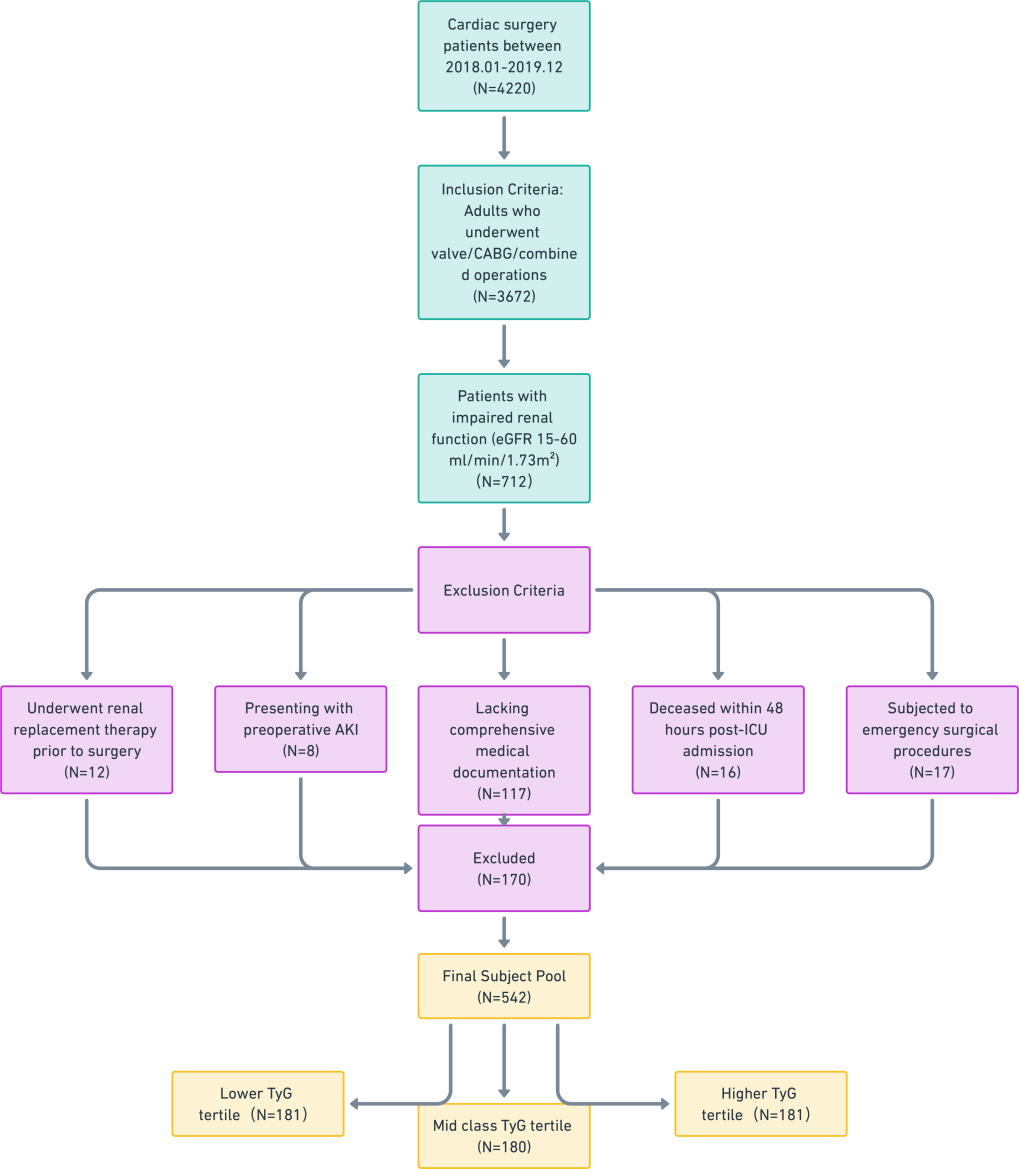
**The Flow chart of Patient Enrollment**. CABG, coronary artery bypass graft; eGFR, estimated glomerular filtration rate; AKI, acute kidney injury; ICU, intensive care unit; TyG, triglyceride-glucose. Fig. 1 was created by Whimsical (https://whimsical.com/).

The patients enrolled into this study met following inclusion criteria (shown in Fig. 1):

(a) Adults aged ≥18 years.

(b) Diagnosed with CKD based on an estimated glomerular filtration rate (eGFR) of 15–60 mL/min/1.73 m^2^ (Chronic Kidney Disease Epidemiology Collaboration (CKD-EPI) formula).

(c) Underwent elective valve, coronary artery bypass, or combined surgery.

Those patients who met following exclusion criteria were excluded:

(a) Preoperative renal replacement therapy.

(b) Preoperative AKI.

(c) Emergency surgical procedures.

(d) Incomplete clinical data.

(e) Death within 48 hours post-intensive care unit (ICU) admission.

Although informed consent is not always mandatory for retrospective observational studies, this study is part of a broader research series involving cardiac surgery patients, some of which include interventional studies requiring informed consent. Therefore, informed consent was obtained from all participants at the time of their hospital admission. This consent covered the use of their clinical data for retrospective and prospective research purposes, ensuring qualification for any future studies involving interventions. Ethical approval was obtained from the Zhongshan Hospital Ethics Committee, and all eligible participants provided written informed consent.

### 2.2 Study Design

In this retrospective study, clinical data were obtained from electronic health records, encompassing patient demographics, pre-existing conditions, laboratory findings, surgical details, duration of cardiopulmonary bypass (CPB), post-surgical medication, urine output, duration of ICU/hospital stay, and mortality. CKD was diagnosed based on an eGFR less than 60 mL/min/1.73 m^2^ using the CKD-EPI formula [18], based on the most recent pre-surgical serum creatinine measurements, and was used to estimate glomerular filtration rates. Preoperative renal function assessment was performed within three days prior to surgery to ensure accurate preoperative classification.

The primary endpoint was the incidence of AKI, defined using the Kidney Disease: Improving Global Outcomes (KDIGO) criteria [19]. Serum creatinine was measured using an enzymatic assay in the hospital’s central laboratory, which undergoes regular quality control to ensure accuracy and precision. Daily serum creatinine measurements were obtained during the ICU stay, with additional measurements made every three days following ICU discharge and then every other day until hospital discharge. Efforts were made to minimize potential errors, including standardizing pre-surgical and post-surgical sample collection times and ensuring consistent use of laboratory equipment and reagents. These protocols were designed to align with the KDIGO criteria and ensure a reliable AKI diagnosis. Secondary outcomes included in-hospital mortality and length of hospital stay. In-hospital mortality was defined as death from any cause occurring during the hospitalization period, starting 48 hours post-surgery, while length of hospital stay was defined as the total duration from admission to discharge or death, encompassing both preoperative and postoperative periods.

The TyG index was calculated using the following formula: Ln(fasting triglycerides (TG) [mg/dL] × FBG [mg/dL]/2). All patients were divided into three groups according to TyG tertile. Namely, group T1 (7.29 ≤ TyG < 8.41), group T2 (8.41 ≤ TyG < 8.96), and group T3 (8.96 ≤ TyG ≤ 11.9).

### 2.3 Selection of Covariates

Our study employed several covariates to control for potential confounding factors, including age, sex, body mass index (BMI), pre-operative comorbidities, baseline cardiac function (classified by New York Heart Association (NYHA) stage), laboratory indices, surgical type, and CPB duration. These variables were selected based on literature evidence [2, 20] linking them to AKI risk and their clinical relevance in cardiac surgery patients.

### 2.4 Statistical Analysis

In our study, all statistical analyses were performed using R software , version 4.2.2 (R Foundation for Statistical Computing, Vienna, Austria). Normally distributed data were depicted as the mean ± standard deviation, non-normally distributed continuous variables as medians with interquartile ranges, and categorical variables as counts and percentages. Normality and variance homogeneity assessment were employed using the Kolmogorov-Smirnov test. To analyze the differences between participants categorized by TyG tertiles, the Student *t*-test and nonparametric tests were used to determine differences in continuous data, while analysis of categorical variables were performed using the Fisher’s exact or chi-square tests. Due to the non-normal distribution of TyG, natural logarithm (Ln) transformations were applied. Multivariable logistic regression models were used to examine the associations between Ln-transformed/standardized TyG and endpoints. Sensitivity analyses included combining the Ln-transformed TyG with a standardized TyG index and examining crude results to validate the accuracy of the data. Multivariable logistic regression models, ranging from Model 1 to Model 3, examined the association between TyG and AKI/in-hospital mortality/length of hospital stay, adjusting for various confounders. In Model 1 no covariates was adjusted; Model 2 adjusted for age, sex, BMI, surgical type, hypertension, diabetes, and NYHA class; Model 3 adjusted for age, sex, BMI, surgical type, hypertension, diabetes, NYHA class, pre-operative albumin, pre-operative hemoglobin, and pre-operative serum creatinine. Subgroup analyses were conducted to explore the associations between TyG and endpoints among individuals of different sexes, ages (≥65 vs. <65 years), BMI (≥24 vs. <24 kg/m^2^), diabetes status, hypertension status, surgical type, eGFR class and NYHA class. Interaction tests determined the consistency of these associations across the various subgroups. The number of each subgroup were reported to ensure sufficient statistical power for interaction tests. The significance threshold was established at *p* < 0.05.

## 3. Results

### 3.1 Baseline Characteristics of Entire Cohort

Among the 542 eligible participants in our analysis, 55.7% developed AKI after cardiac surgery. The in-hospital mortality of the entire cohort was 7.6%. As shown in Table 1, there was no significance differences in the prevalence of AKI, renal replacement therapy (RRT), and in-hospital mortality across the TyG tertiles (all *p* > 0.05).

**Table 1.** **Patient demographics and baseline characteristics**.

Characteristic		TyG tertiles			*p*-value
		(7.29, 8.41), N = 181	(8.41, 8.96), N = 180	(8.96, 11.9), N = 181	
Age (yrs)		67 (60, 74)	66 (60, 72)	65 (60, 72)	0.249
Male		103 (56.9%)	108 (60.0%)	112 (61.9%)	0.623
BMI (kg/m^2^)		22.6 (20.0, 24.2)	22.8 (22.2, 25.9)	23.3 (22.6, 26.3)	<0.001
Hypertension		83 (45.9%)	88 (48.9%)	129 (71.3%)	<0.001
Diabetes		17 (9.4%)	37 (20.6%)	53 (29.3%)	<0.001
NYHA >2		119 (65.7%)	101 (56.1%)	98 (54.1%)	0.056
RAASi		121 (66.8%)	114 (63.3%)	124 (68.5%)	0.569
Statins		76 (41.9%)	82 (45.5%)	83 (45.9%)	0.712
Pre-operative laboratory indices					
	BUN (mmol/L)	9.0 (7.0, 11.0)	9.0 (7.0, 11.0)	9.0 (7.0, 11.0)	0.462
	Creatinine (µmol/L)	120 (103, 140)	122 (103, 135)	125 (112, 142)	0.040
	UA (mmol/L)	437 (371, 559)	464 (373, 558)	514 (409, 601)	0.002
	eGFR (mL/min/1.73 m^2^)	52 (45, 56)	52 (47, 56)	50 (42, 56)	0.188
	Hemoglobin (g/L)	126 ± 17	129 ± 17	128 ± 18	0.237
	Albumin (g/L)	39.0 (36.0, 41.0)	40.0 (37.0, 42.0)	40.0 (38.0, 42.0)	0.006
	Blood glucose (mmol/L)	4.70 (4.40, 5.20)	5.20 (4.80, 5.63)	6.10 (5.40, 8.40)	<0.001
	TC (mmol/L)	3.91 (3.29, 4.51)	4.22 (3.52, 4.83)	4.39 (3.73, 5.00)	<0.001
	TG (mmol/L)	0.91 (0.77, 1.04)	1.37 (1.24, 1.55)	2.22 (1.79, 3.03)	<0.001
	LDL-C (mmol/L)	2.31 (1.70, 2.75)	2.44 (1.91, 3.03)	2.36 (1.69, 3.00)	0.161
	HDL-C (mmol/L)	1.19 (1.00, 1.44)	1.03 (0.89, 1.25)	0.94 (0.80, 1.08)	<0.001
Surgical type					<0.001
	CABG	58 (32.0%)	78 (43.3%)	97 (53.6%)	
	Valve	109 (60.2%)	86 (47.8%)	66 (36.5%)	
	Valve+CABG	14 (7.8%)	16 (8.9%)	18 (9.9%)	
CPB duration (mins)		94 (77, 127)	97 (79, 129)	101 (79, 129)	0.664
ACC duration (mins)		56 (40, 74)	53 (40, 77)	56 (46, 77)	0.362
AKI stages					0.233
	1	67 (37.0%)	54 (30.0%)	69 (38.1%)	
	2	25 (13.8%)	26 (14.4%)	16 (8.8%)	
	3	15 (8.3%)	18 (10.0%)	12 (6.6%)	
RRT		10 (5.5%)	8 (4.4%)	9 (5.0%)	0.895
In-hospital mortality		14 (7.7%)	18 (10.0%)	9 (5.0%)	0.195
LOS (days)		16 (12, 22)	14 (12, 21)	16 (12, 22)	0.317
Cost (USD)		12,653 (8821, 17,443)	12,193 (9072, 16,791)	12,810 (9951, 16,699)	0.582

ACC, aortic cross clamp; AKI, acute kidney injury; BMI, body mass index; BUN, blood urea nitrogen; CABG, coronary artery bypass graft; CPB, cardiopulmonary bypass; eGFR, estimated glomerular filtration rate; HDL-C, high-density lipoprotein cholesterol; LDL-C, low-density lipoprotein cholesterol; LOS, length of stay; NYHA, New York Heart Association; RAASi, renin-angiotensin-aldosterone system inhibitors; RRT, renal replacement therapy; TC, total cholesterol; TG, triglycerides; TyG, triglyceride-glucose; UA, uric acid.

Numerous variables, such as BMI, hypertension, diabetes, serum creatinine, uric acid, albumin, blood glucose, total cholesterol, triglyceride, high-density lipoprotein cholesterol (HDL-C) and surgical type showed significant differences across TyG tertiles (all *p* < 0.05).

### 3.2 Association Between TyG and Endpoints

Table 2 displayed the association between TyG with AKI, in-hospital mortality and length of hospital stay. After adjusting for covariates, no significant association was found between TyG with AKI, in-hospital mortality and length of hospital stay.

**Table 2.** **Association between TyG and endpoints**.

Characteristic		Model 1			Model 2			Model 3		
		OR (HR)	95% CI	*p*-value	OR (HR)	95% CI	*p*-value	OR (HR)	95% CI	*p*-value
AKI (Logistic regression)										
LnTyG (continuous)		1.32	0.13, 13.13	0.814	0.45	0.03, 7.01	0.567	0.43	0.02, 8.70	0.579
TyG (standardized)		1.03	0.87, 1.22	0.718	0.96	0.77, 1.20	0.707	0.96	0.77, 1.21	0.754
TyG										
	L [7.29, 8.41)	—	—		—	—		—	—	
	M [8.41, 8.96)	0.80	0.49, 1.29	0.362	0.81	0.48, 1.34	0.411	0.83	0.50, 1.39	0.479
	H [8.96, 11.9]	0.87	0.54, 1.42	0.587	0.61	0.36, 1.03	0.067	0.62	0.36, 1.05	0.079
In-hospital mortality (Cox regression)										
LnTyG (continuous)		0.38	0.01, 25.3	0.649	0.16	0.00, 34.94	0.504	0.17	0.00, 38.50	0.520
TyG (standardized)		0.93	0.68, 1.28	0.676	0.84	0.56, 1.27	0.407	0.85	0.57, 1.28	0.440
TyG										
	L [7.29, 8.41)	—	—		—	—		—	—	
	M [8.41, 8.96)	1.63	0.81, 3.29	0.174	1.24	0.53, 2.90	0.620	1.30	0.55, 3.08	0.546
	H [8.96, 11.9]	0.60	0.25, 1.42	0.242	0.37	0.11, 1.08	0.078	0.35	0.10, 1.04	0.067
Length of hospital stay (Linear regression)										
LnTyG (continuous)		–1.32	–16.49, 13.85	0.865	8.29	–9.21, 25.78	0.354	–2.58	–20.88, 15.72	0.782
TyG (standardized)		–0.01	–0.09, 0.08	0.887	0.01	–0.10, 0.11	0.899	–0.01	–0.11, 0.09	0.871
TyG										
	L [7.29, 8.41)	—	—		—	—		—	—	
	M [8.41, 8.96)	–1.65	–4.39, 1.09	0.239	–1.55	–4.68, 1.58	0.332	–1.58	–4.65, 1.50	0.316
	H [8.96, 11.9]	0.22	–2.52, 2.95	0.877	1.01	–2.14, 4.15	0.531	–0.54	–3.74, 2.66	0.740

Model 1 (Crude): no covariates were adjusted. Model 2: adjusted for age, sex, BMI, surgical type, hypertension, diabetes, and NYHA class. Model 3: adjusted for age, sex, BMI, surgical type, hypertension, diabetes, NYHA class, pre-operative albumin, pre-operative hemoglobin, and pre-operative serum creatinine. AKI, acute kidney injury; BMI, body mass index; CI, confidence interval; H, high tertile for TyG index range; HR, hazard ratio; L, low tertile for TyG index range; LnTyG, natural logarithm of triglyceride-glucose; M, middle tertile for TyG index range; NYHA, New York Heart Association; OR, odds ratio; TyG, triglyceride-glucose.

In order to perform a sensitivity analysis, TyG were divided into tertiles. The sensitivity analysis results indicated that the association between TyG index and outcomes (AKI, in-hospital mortality, and length of hospital stay) was not statistically significant across all models.

### 3.3 Subgroup Analysis

**Fig. 2.**
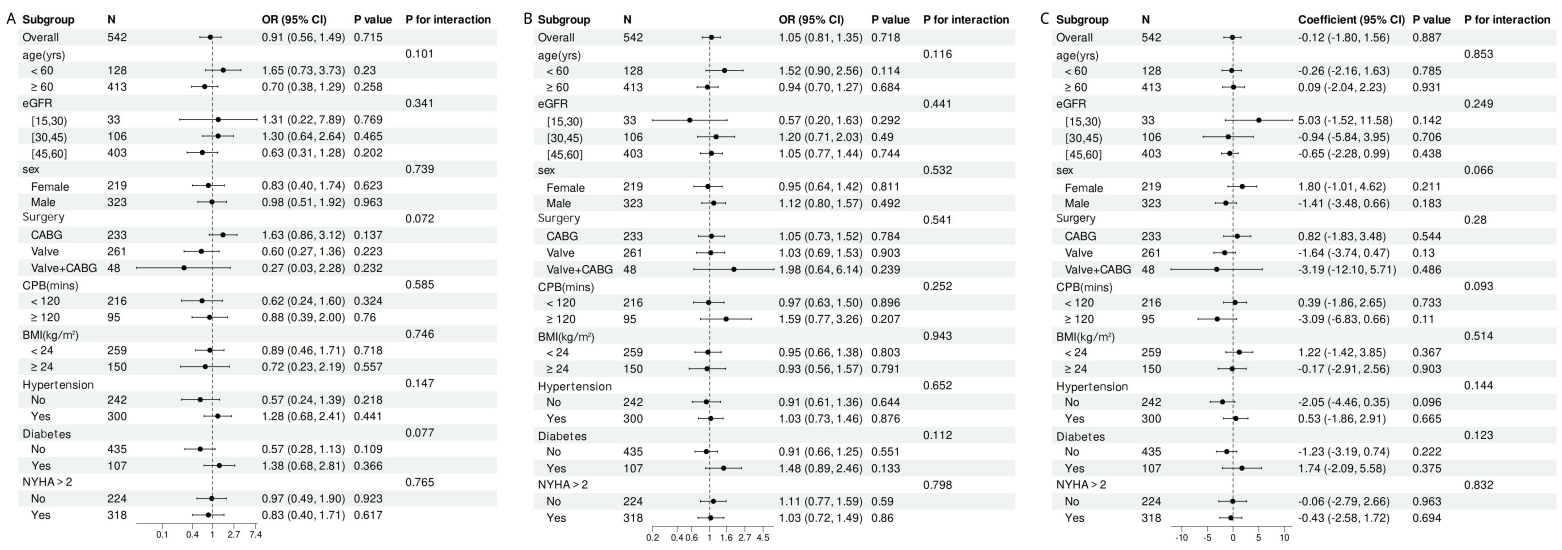
**Subgroup analysis of the association between TyG and endpoints in different patients**. (A) TyG & AKI. (B) TyG & in-hospital mortality. (C) TyG & length of hospital stay. AKI, acute kidney injury; BMI, body mass index; CI, confidence interval; CABG, coronary artery bypass graft; CPB, cardiopulmonary bypass; eGFR, estimated glomerular filtration rate; NYHA, New York Heart Association; OR, odds ratio; TyG, triglyceride-glucose.

Subgroup analyses revealed no significant associations between the TyG index and AKI (Fig. 2A), in-hospital mortality (Fig. 2B), or length of hospital stay (Fig. 2C) across different strata, including age, sex, BMI, and CKD stages. The relationships between TyG with outcomes were not significantly associated in the interaction tests for the different strata (*p* for all interactions >0.05).

## 4. Discussion

Our study sought to evaluate the predictive value of the TyG index for postoperative AKI in patients with preoperative renal insufficiency undergoing cardiac surgery. A recent study proposed the concept of acute-on-chronic kidney injury to define this type of AKI [21]. To comprehensively evaluate potential associations, we performed both sensitivity and subgroup analyses. These analyses, which included alternative TyG stratifications and adjusted regression models, consistently showed no significant association, reinforcing the accuracy of our findings.

In contrast to our findings, several studies have highlighted the potential of the TyG index as a predictive marker for AKI in various patient populations. A cohort study involving 790 patients undergoing coronary revascularization demonstrated a higher risk of AKI with increased TyG index levels [16], suggesting its potential as a predictive tool in these patients.​​ Similarly, a study on critically ill patients with heart failure reported a significant correlation between elevated TyG levels and the incidence of AKI [15], underscoring the index’s utility in predicting renal outcomes in critically ill patient populations​. Research focusing on patients with acute myocardial infarction further supported these findings [17], revealing a strong association between higher TyG levels and AKI, which indicates that TyG might help identify high-risk patients in acute care settings​​. Additionally, a study of critically ill patients with coronary artery disease found that the TyG index could effectively predict AKI, reinforcing the index’s predictive value across different clinical scenarios​​ [22].

The mechanisms linking IR and AKI, particularly in patients with CKD, are complex and multifaceted. Insulin resistance, often exacerbated in CKD, is driven by the activation of the RAAS [23], which leads to increased renal vasoconstriction and sodium retention. This activation results in endothelial dysfunction [24], reducing renal perfusion [25] and increasing the susceptibility to ischemic injury. Furthermore, oxidative stress [26] and inflammation [27], common in insulin resistance, can exacerbate renal tubular damage, promoting the development of AKI​​. During cardiac surgery, these mechanisms are particularly relevant due to the hemodynamic instability, oxidative stress, and inflammatory responses that patients often experience [1, 2], which can further activate RAAS and exacerbate insulin resistance, thereby increasing the risk of AKI.

The lack of a significant association between the TyG index and AKI in our study may be attributable to several factors. First, while insulin resistance has been implicated in renal injury via pathways such as endothelial dysfunction [24], inflammation, and oxidative stress [26], these mechanisms may be less pronounced in CKD patients undergoing cardiac surgery, where direct perioperative risk factors, such as the type of surgery and prolonged duration of CPB, play a dominant role in these pathways. These factors may overshadow the subtler contributions of insulin resistance, rendering the TyG index less predictive in this specific context. Second, CKD patients often exhibit baseline metabolic abnormalities [28] that could attenuate the relative impact of insulin resistance compared to populations without underlying renal dysfunction.

Almost all studies that have found a correlation between the TyG index and AKI have exclusively used the Medical Information Mart for Intensive Care (MIMIC) database. Among these, only one study involved cardiac surgery, specifically CABG. In contrast, our study, based on a Chinese population, includes a significantly higher proportion of more complex valve surgeries. Our study differs from previous MIMIC database-based research, which included 7.9%–27.5% CKD patients [15, 16, 17], by exclusively focusing on a CKD population undergoing cardiac surgery. This distinction is critical, as CKD patients present with unique metabolic and inflammatory profiles that may influence the predictive utility of the TyG index. In addition, while prior study predominantly included patients undergoing CABG [16], our cohort also included valve surgeries (48.15%) and combined valve-CABG procedures (8.8%), reflecting the surgical landscape in Chinese patients. These high-risk procedures and patient characteristics resulted a higher AKI incidence of 55.7%, compared to the 30.13% reported in a prior study.

Despite these differences, our study showed trends similar to previous research, which demonstrated diabetes prevalence, fasting glucose, and lipid levels increasing significantly across TyG tertiles (shown in Table 1). The consistency in AKI definitions (KDIGO criteria) and TyG calculation methods further supports the reliability of our findings. Therefore, we attribute the observed differences in outcomes primarily to the distinct characteristics of our study population and surgical procedures. Consequently, the insulin resistance mechanism represented by the TyG index may be overshadowed by more direct risk factors such as cardiopulmonary bypass. Using the Boruta algorithm, which is an all-relevant feature selection method known for identifying important predictors by comparing the importance of original attributes with randomized versions, we discovered that only the duration of cardiopulmonary bypass emerged as a significant predictor of AKI. In contrast, the TyG index was not identified as an important factor (**Supplementary Fig. 1**).

In our analysis, the use of RAAS inhibitors and statins was considered due to their known effects on metabolic and cardiovascular outcomes, which could potentially confound our data analysis. Although the proportional use of these medications did not differ significantly across the TyG index tertiles as detailed in Table 1, it is important to acknowledge that these drugs could influence the TyG index and AKI outcomes independently of the statistical distributions we observed. RAAS inhibitors can modify renal hemodynamics and therefore could impact the incidence of AKI, while statins may affect insulin resistance and lipid metabolism, thus altering the TyG index. The similar usage rates across groups suggest that the influence of these medications on our findings might be evenly distributed among the cohorts, reducing the likelihood that they skewed the associations between the TyG index and postoperative outcomes.

Our study has several limitations. First, the observational design inherently limits the ability to establish causality, and the lack of randomization may introduce selection bias. The findings are based on data from a single center, which may limit generalizability to other settings or populations. Despite adjustments for various confounders, unmeasured factors might influence the observed associations. Moreover, the patients did not have their postoperative lipid profiles measured, making it impossible to compare perioperative changes in the TyG index and analyze its association with AKI. Another limitation of this study is the lack of detailed data on specific intraoperative factors, such as the type of anesthesia used, which could potentially influence the outcomes. Future studies should aim to include these variables to provide a more comprehensive analysis. Our failure of collecting the history of identifying type 1 and type 2 diabetes in our cohort, may also have confounded our results given the distinct pathophysiological mechanisms and management strategies associated with each type. Furthermore, we were unable to access and analyze emerging biomarkers of AKI such as neutrophil gelatinase-associated lipocalin, kidney injury molecule-1, and cystatin C. The inclusion of these biomarkers could have provided a more comprehensive understanding of the pathophysiological changes and risk stratification in our patient population, potentially enhancing the predictive accuracy of our findings. While our findings suggested that the TyG index may have limited predictive value in this specific population, the possibility of an association cannot be entirely ruled out. Future studies with larger sample sizes may help to clarify whether a more subtle relationship exists between TyG and AKI, as well as its long-term prognostic implications.

## 5. Conclusions

This study found no significant association between the preoperative TyG index and postoperative AKI, in-hospital mortality, or length of hospital stay in patients with preoperative renal insufficiency undergoing cardiac surgery. These findings suggest that the TyG index may have limited utility in predicting these specific outcomes in this patient population. However, this does not preclude the broader relevance of insulin resistance in other contexts, and further research is needed to explore its role in perioperative outcomes using alternative markers and in different patient cohorts.

## Data Availability

The datasets used and/or analyzed during the current study are available from the corresponding author on reasonable request.

## Abbreviations

AKI, acute kidney injury; BMI, body mass index; CABG, coronary artery bypass grafting; CKD, chronic kidney disease; CKD-EPI, Chronic Kidney Disease Epidemiology Collaboration; CPB, cardiopulmonary bypass; eGFR, estimated glomerular filtration rate; ICU, intensive care unit; IR, insulin resistance; KDIGO, Kidney Disease: Improving Global Outcomes; NYHA, New York Heart Association; RAAS, renin-angiotensin-aldosterone system; RRT, renal replacement therapy.

## Supplementary Material

Supplementary material associated with this article can be found, in the online version, at https://doi.org/10.31083/RCM28110.
