# Supplementary figures and images for: Association Between the Preoperative Triglyceride–Glucose Index and Acute Kidney Injury in Patients With Chronic Kidney Disease Undergoing Cardiac Surgery
Source: Rev Cardiovasc Med. 2025 Jun 17;26(6):28110. doi: 10.31083/RCM28110 (PMC12230818; doi:10.31083/RCM28110)

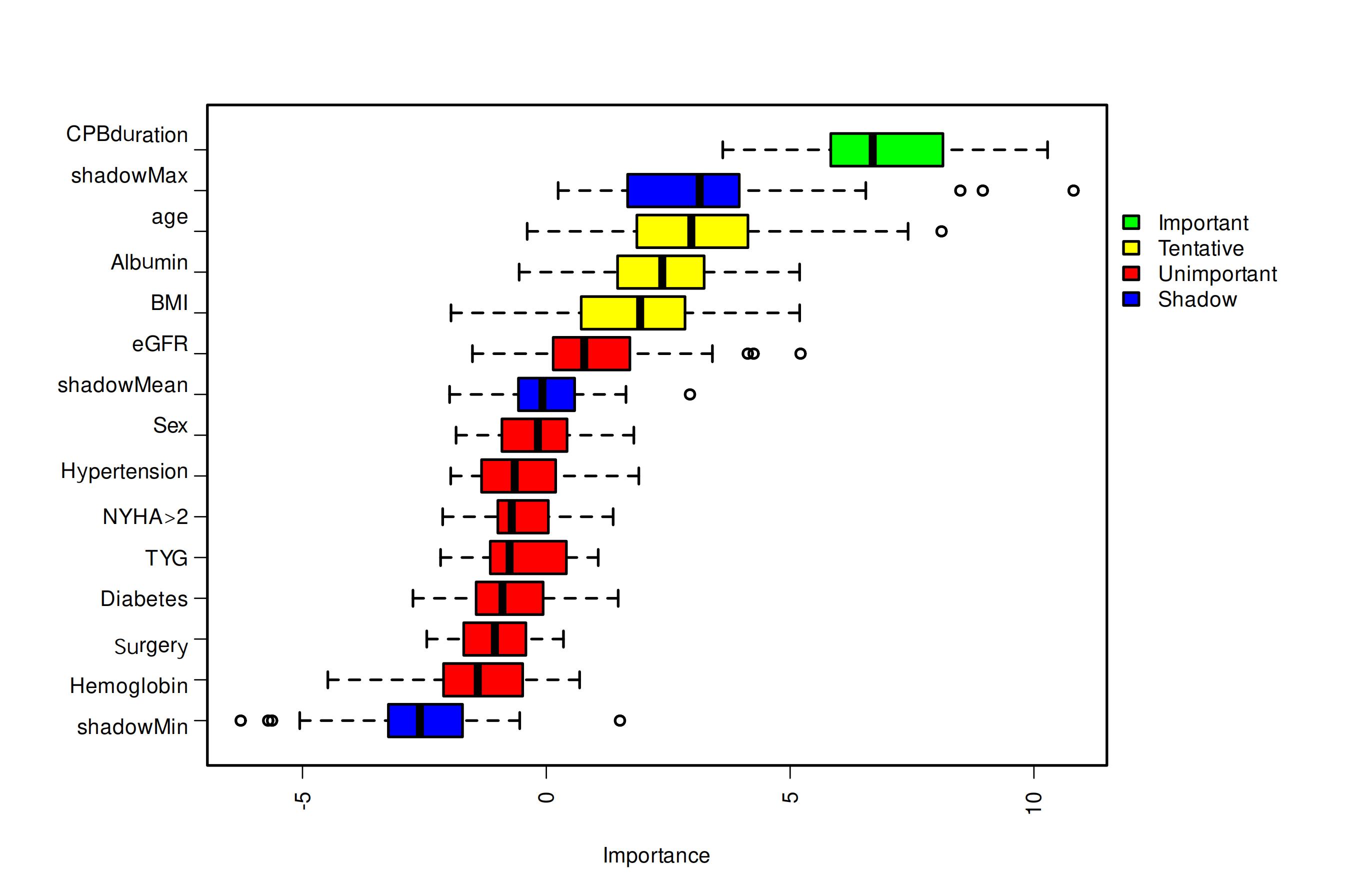

Supplement: Supplementary file 1 [file 2153-8174-26-6-28110-s1.zip › Supplementary Fig.1.jpg]
